# Breath Fingerprint of Colorectal Cancer Patients Based on the Gas Chromatography–Mass Spectrometry Analysis

**DOI:** 10.3390/ijms25031632

**Published:** 2024-01-29

**Authors:** Elīna Kononova, Linda Mežmale, Inese Poļaka, Viktors Veliks, Linda Anarkulova, Ilona Vilkoite, Ivars Tolmanis, Anna Marija Ļeščinska, Ilmārs Stonāns, Andrejs Pčolkins, Pawel Mochalski, Mārcis Leja

**Affiliations:** 1 Institute of Clinical and Preventive Medicine, University of Latvia, LV-1586 Riga, Latvia; elina.kononova@lu.lv (E.K.); inese.polaka@lu.lv (I.P.); viktors.veliks@lu.lv (V.V.); linda.anarkulova@gmail.com (L.A.); lescinska27@gmail.com (A.M.Ļ.); ilmars.stonans@lu.lv (I.S.); andrejs.pcholkins@gmail.com (A.P.); marcis.leja@lu.lv (M.L.); 2 Faculty of Medicine, Riga Stradins University, LV-1007 Riga, Latvia; ivars.tolmanis@gastrocentrs.lv; 3 Riga East University Hospital, LV-1038 Riga, Latvia; 4 Health Centre 4, LV-1012 Riga, Latvia; ilona153@inbox.lv; 5 Department of Modelling and Simulation, Riga Technical University, LV-1048 Riga, Latvia; 6 Liepaja Regional Hospital, LV-3414 Liepaja, Latvia; 7 Department of Doctoral Studies, Riga Stradins University, LV-1007 Riga, Latvia; 8 Digestive Diseases Centre GASTRO, LV-1079 Riga, Latvia; 9 Faculty of Medicine, University of Latvia, LV-1586 Riga, Latvia; 10 Institute for Breath Research, University of Innsbruck, 6020 Innsbruck, Austria; pawel.mochalski@uibk.ac.at; 11 Institute of Chemistry, Jan Kochanowski University of Kielce, 25-369 Kielce, Poland

**Keywords:** colorectal cancer, GC-MS, volatile organic compounds, VOCs, CRC, breath test, CRC marker, screening

## Abstract

The human body emits a multitude of volatile organic compounds (VOCs) via tissues and various bodily fluids or exhaled breath. These compounds collectively create a distinctive chemical profile, which can potentially be employed to identify changes in human metabolism associated with colorectal cancer (CRC) and, consequently, facilitate the diagnosis of this disease. The main goal of this study was to investigate and characterize the VOCs’ chemical patterns associated with the breath of CRC patients and controls and identify potential expiratory markers of this disease. For this purpose, gas chromatography–mass spectrometry was applied. Collectively, 1656 distinct compounds were identified in the breath samples provided by 152 subjects. Twenty-two statistically significant VOCs (p-xylene; hexanal; 2-methyl-1,3-dioxolane; 2,2,4-trimethyl-1,3-pentanediol diisobutyrate; hexadecane; nonane; ethylbenzene; cyclohexanone; diethyl phthalate; 6-methyl-5-hepten-2-one; tetrahydro-2H-pyran-2-one; 2-butanone; benzaldehyde; dodecanal; benzothiazole; tetradecane; 1-dodecanol; 1-benzene; 3-methylcyclopentyl acetate; 1-nonene; toluene) were observed at higher concentrations in the exhaled breath of the CRC group. The elevated levels of these VOCs in CRC patients’ breath suggest the potential for these compounds to serve as biomarkers for CRC.

## 1. Introduction

In the year 2020, colorectal cancer (CRC) stood as the third most diagnosed cancer globally and the second principal cause of cancer-related mortality [1]. In that year, estimations indicated over 1.9 million new cases of CRC and approximately 935,000 related fatalities [1]. The 5-year survival rate for CRC can soar to 90% when the condition is detected in its early stages, significantly elevating the chances of a favorable outcome. Early detection is crucial in improving patient outcomes and reducing mortality rates, making it a priority in any national health system initiative to develop a dependable and efficient screening tool.

CRC screening modalities do exist and are recommended for routine clinical applications in most developed countries, but there is still definite room for improvement. Presently, two non-invasive screening methods are used: a stool-based test that detects occult blood, namely the guaiac-based fecal occult blood test (gFOBT), and the fecal immunochemical test (FIT). FIT is more sensitive than gFOBT in detecting precancerous lesions and cancer, leading to a strong recommendation for prioritizing FIT over gFOBT [2,3,4,5]. Although FIT is currently the non-invasive test of choice, variability in cutoff levels for positive results leads to inconsistencies in diagnosis and complicates the establishment of a clinical standard [6,7]. Additionally, FIT is not perfectly sensitive, especially in detecting adenomas [8]. Despite the availability of CRC screening methods, participation in public screening programs is low due to psychological or physical discomfort associated with the tests and the need for improved accuracy. Colonoscopy is considered the gold standard, serving dual purposes as both a primary screening tool and a follow-up procedure for individuals who have tested positive through other screening methods [9,10]. While highly sensitive, it is time- and resource-demanding and may cause complications such as bowel perforation, bleeding, dehydration due to bowel preparation, and cardiovascular events due to sedation [11,12].

Newer screening techniques include multitarget stool DNA testing (FIT-DNA), which combines FIT with the analysis of altered DNA biomarkers in stool cells. This approach has a significantly higher cancer detection rate compared to FIT alone but falls short in terms of specificity, potentially leading to an increased number of unnecessary colonoscopies [13]. Serology tests designed to identify circulating methylated SEPT9 DNA present comparatively lower sensitivity, with a standardized sensitivity of 48.2% [14].

These limitations underscore the need for an alternative non-invasive, cost-effective, low-risk, and highly sensitive screening test to prevent overdiagnosis. One promising method involves the use of volatile organic compounds (VOCs) in exhaled breath, which has shown encouraging results [15,16,17,18,19]. The concept is based on the premise that VOCs in human breath are indicators of metabolic processes and diseases. These compounds, identifiable in various biological substances including tissues, urine, blood, and, notably, exhaled breath, suggest that cancer cells release VOCs into the bloodstream. Subsequently, these compounds are excreted through the lungs and can be detected in exhaled air [20,21,22].

Investigating cancer-specific VOCs in biological fluids is a promising research direction. Previous studies have identified cancer-related VOCs in patients with various types of cancer, including stomach, breast, lung, and CRC [23,24,25,26,27]. Techniques such as gas chromatography–mass spectrometry (GC-MS), ion mobility spectrometry (IMS), proton transfer reaction mass spectrometry (PTR-MS), and electronic nose (e-nose) have been employed for analysis [28]. Numerous studies exploring the viability of VOC analysis as a CRC screening tool have yielded promising outcomes [15,29].

The main goal of this study was to investigate and characterize the VOC chemical patterns associated with the breath of CRC patients and to identify potential expiratory markers of this disease.

## 2. Results

In total, the study encompassed a total of 78 patients diagnosed with CRC and 74 individuals serving as controls; the median age of the study subjects being 63. Notably, within this cohort, analysis revealed no statistically significant disparity in the prevalence of CRC between genders, as indicated by a *p*-value of 0.836.

The CRC cohort exhibited diverse clinical stages and varying degrees of cancer differentiation in colorectal adenocarcinoma. A comprehensive account of the clinical features of the participants is outlined in Table 1 for a more detailed insight into the distinct characteristics observed within the study group.

Altogether, the analysis of breath samples from 152 subjects revealed the presence of 1656 distinct compounds. Among these, 1210 were identified in samples obtained from CRC patients, and 1267 were found in samples provided by control subjects. 

The VOCs, systematically categorized based on their chemical classes and exhibiting an incidence rate exceeding 50% are outlined in Table 2, offering a detailed representation of the prevalent constituents in the analyzed breath samples. Within both groups, aldehydes, esters, and ketones emerged as the predominant classes of VOCs. These were succeeded by hydrocarbons, alcohols, aromatics, and heterocycles. The variability in the number of identified compounds per sample was evident, with subjects exhibiting a range from 50 to 93. This observation underscores the diverse composition of VOCs among individuals, emphasizing the intricate nature of the analyzed samples.

The distribution of VOCs based on their chemical classes was similar in the two groups under study. Table 3 lists the compounds with an occurrence exceeding 30%. In both patients and controls, the distribution of VOCs was comparable, with aromatics being the dominant class, comprising 12 compounds. Out of the 1656 compounds detected, we selected 21 statistically significant VOCs that were observed at higher concentrations in the exhaled breath of the CRC group compared to the controls. These compounds are listed in Table 3. The VOCs are ordered according to increasing *p*-values from the Wilcoxon rank-sum test, and only those compounds where the difference between groups was statistically significant are included. As indicated by the data, the levels of all these compounds were higher in the cancer group. Additional identified VOCs can be found in the Supplementary Information, Table S1. 

**Figure 1:**
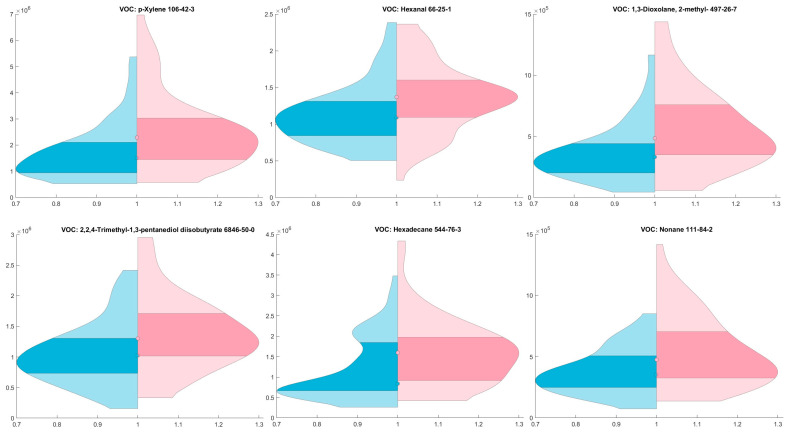
Comparison of Chemical Spike Areas Between Control and Cancer Groups: Violin Plot Analysis; blue represents the control group, while red represents the cancer group; Y-axis denotes the area values, with dots indicating the median for each group and the darker area indicates 25th to 75th percentile. Figure shows the most common VOCs in groups.

The violin plot (Figure 1) illustrates the distribution of chemical spike areas across two distinct group—a control group and cancer group.

## 3. Discussion

The current study elucidates significant differences in the breath VOC patterns between CRC patients and healthy controls, identifying 21 statistically significant VOCs (p-xylene; hexanal; 2-methyl-1,3-Dioxolane; 2,2,4-trimethyl-1,3-pentanediol diisobutyrate; hexadecane; nonane; ethylbenzene; cyclohexanone; diethyl phthalate; 6-methyl-5-hepten-2-one; tetrahydro-2H-pyran-2-one; 2-butanone; benzaldehyde; dodecanal; benzothiazole; tetradecane; 1-dodecanol; 1-benzene; 3-methylcyclopentyl acetate; 1-nonene; toluene). These compounds, that include a range of aldehydes, hydrocarbons, and aromatic compounds, not only differentiate CRC patients from healthy individuals but also offer insights into the underlying metabolic and biochemical changes induced by CRC.

Each compound may uniquely contribute to the overall breath fingerprint, reflecting specific metabolic and biochemical changes in CRC, such as changes in lipid metabolism and increased oxidative stress. For example, higher levels of aldehydes, such as hexanal, might result from cell membrane fatty acid peroxidation due to reactive oxygen species (ROS) [30,31]. Ketones, such as cyclohexanone and 2-butanone, are formed because of increased fatty acid oxidation [32]. Aromatic compounds, such as toluene and benzene, are commonly associated with the breakdown of cellular components and could reflect the increased cell turnover in cancer [33].

However, it is important to note that there is no consensus yet on the most prevalent VOCs in CRC patients’ breath, and research is expanding to other biological materials like urine, blood, feces, and cancer tissues [20,33,34,35]. Studies in these areas, such as Wen Qing et al.’s research on urinary VOCs in cancer, have identified different predominant VOCs, suggesting that the VOC profile might vary with the biological material examined [33]. In contrast, our previous study, which focused on comparing VOCs released in cancerous versus non-cancerous tissues, revealed a predominance of hydrocarbons and alcohols, with aldehydes, ketones, and aromatic compounds following in prevalence [20].

Altomare et al.’s research, focusing on breath VOCs with high discriminant power for CRC, identified key VOCs (tetradecane, ethylbenzene, and benzaldehyde) that overlap with those found in our study, lending further credibility to these compounds as potential biomarkers [17]. Additionally, Śmiełowska et al.’s study on both breath and fecal samples from CRC patients demonstrates not only the diversity in VOCs but also the differences in their concentrations across different sample types, indicating that the VOC profile might be more pronounced in breath samples [36].

Wang Changsong et al. [37] identified cyclohexanone as a notable VOC in CRC patients’ breath, which is consistent with our findings. In our study, along with cyclohexanone, we also detected tetradecane, dodecanal, and 2-butanone. These biomarkers were previously identified in our research on VOC emissions from cancerous versus normal colon tissues, though in that study these four compounds were found at reduced concentrations in cancerous tissue compared to healthy tissue [20]. This discrepancy calls for further investigation, taking into account factors such as the mixing of room air VOCs with exhaled air, the solubility of VOCs in blood, and other variables [38].

The detection of VOCs like hexanal, linked to lung cancer, and 2-butanone, associated with lung and breast cancer, highlights the broader implications of VOCs in cancer detection [39]. This underscores the importance of understanding the complex nature of VOC profiles, which vary significantly in health and disease [40,41].

Our research adds to the existing knowledge base by introducing new VOCs and corroborating the findings of similar VOCs in CRC from other studies. However, the challenge remains in distinguishing cancer-specific VOCs or groups due to their diverse chemical nature and varying presence. Factors like gut microbiota, diet, and other health conditions can influence VOC profiles, complicating their interpretation [42].

Several limitations of this study should be mentioned. Firstly, the relatively small sample size of 152 individuals, coupled with the recruitment of control participants from one clinic and CRC patients from a single hospital in the same city, while sufficient for initial analysis, raises concerns about the geographical and genetic diversity of breath profiles and the broader applicability of our findings. Secondly, another significant limitation is the lack of consideration for the stage of CRC in the analysis of VOCs. The absence of detailed data on the proportion of patients with late-stage versus early-stage adenocarcinoma limits our ability to draw comprehensive conclusions about the disease stages. Lastly, the way samples were stored and processed could have affected the chemical patterns that were observed. Despite these challenges, the exploration of CRC patients’ breath fingerprint through GC-MS analysis is an innovative approach with significant potential in cancer diagnostics. While there are limitations, the promising results and the non-invasive nature of this method make it an exciting area for future research and development in oncology.

## 4. Materials and Methods

### 4.1. Chemical Standards and Quality Benchmarks

All reference mixtures were generated using high-purity liquid chemicals with stated purities ranging from 95% to 99.9%, sourced from Merck (Wien, Austria). The preparation of standards involved a two-step process. Initially, a few microliters of a liquid compound were introduced into evacuated and heated 1 L glass bulbs (Supelco, Toronto, Canada) to produce primary standards. Once the compounds had evaporated, the bulb pressure was equalized using nitrogen. Subsequently, the primary standards were diluted by transferring precise volumes from the bulb mixtures into 3–25 L Tedlar bags (SKC Inc., Eighty Four, PA, USA). These bags had been prefilled with purified and humidified air (with a relative humidity of 100% at 34 °C). The standards were sampled within a 30 min time window after production.

We acquired stainless steel industry-standard thermal desorption tubes (1/4 inch outer diameter, 3½ inches long) from Markes International (Bridgend, UK). These tubes were prefilled with Tenax TA (Bridgend, UK) and coated with SilcoNert™ (Bellefonte, PA, USA). Before each sampling event, the sorbent tubes underwent reconditioning procedures following the manufacturer’s guidelines.

### 4.2. Study Group Description and Recruitment Process

The study enrolled individuals diagnosed with CRC based on confirmed morphological assessments, including patients with confirmed adenocarcinoma, who donated a breath sample before undergoing surgical treatment. The control cohort consisted of individuals without high-risk precancerous lesions or colorectal adenocarcinoma, all of whom had undergone a colonoscopy. The definitive categorization for the study was determined following the examination of the morphological report.

Participants were sourced from two medical facilities: the Riga East Clinical University Hospital within the Oncology Center of Latvia and the Digestive Diseases Centre GASTRO in Riga, Latvia.

The enrollment criteria included individuals aged 18 and above who provided signed consent forms. Exclusion criteria were established to minimize potential confounding factors from other medical conditions. Individuals with concurrent active malignancies, a history of complete bowel cleansing, inflammatory bowel diseases, previous bowel resection, ongoing neoadjuvant chemotherapy and/or radiation therapy, acute conditions requiring emergency surgery, chronic renal failure stage 4, type I diabetes, and active bronchial asthma were excluded.

### 4.3. Breath Sample Collection

Breath samples were taken in a designated room that was free from any chemicals, cleaning agents, medications, solvents, or kitchen waste. Samples were taken at room temperature.

To reduce the impact of possible variables that could interfere with the accuracy of exhaled breath analysis, participants were provided with clear guidelines. They were instructed to adhere to certain practices, including fasting overnight, refraining from smoking and alcohol consumption, avoiding gum chewing, and abstaining from physical activity for at least two hours before providing breath samples. Furthermore, participants were advised not to use perfume until after the collection of their breath samples. These measures were implemented to ensure the reliability and integrity of the collected breath samples for analysis.

**Figure 2:**
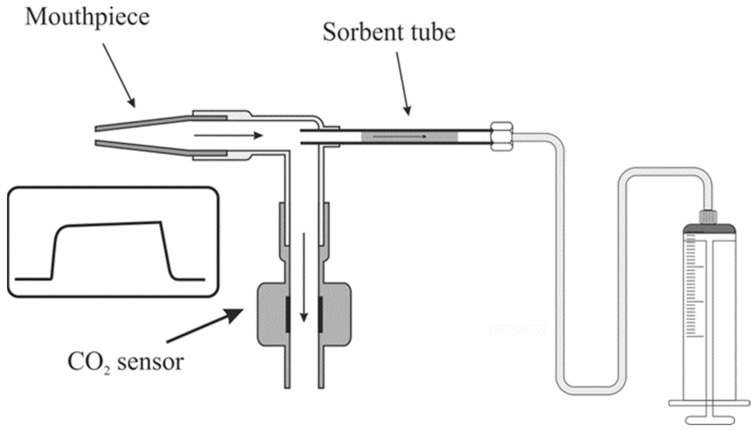
Scheme of sampling system used in the study and consisting of mouthpiece, CO_2_ control sensor, sorbent tube, and a syringe for drawing air.

Breath samples were collected using a custom-designed breath sampler illustrated in Figure 2. This sampler comprised a single-use mouthpiece (Intersurgical) attached to a disposable elbow (Intersurgical) and the CO_2_ sensor cell (Masimo, Irvine, CA, USA; IRMA, Seattle, WA, USA) connected to the opposite end of the elbow. The elbow featured a 1/4” port, facilitating the attachment of industry-standard ¼” sorbent tubes. Directly before sampling, the sampling end of a sorbent tube was inserted into the elbow so that it protruded 5–6 mm into its interior and secured with a ¼” PTFE nut. The other end of the sampling tube was connected to a 250 mL glass syringe (Socorex, Switzerland) using a 1/8” Teflon (Wilmington, DE, USA) tube. Participants could freely inhale/exhale through a mouthpiece without encountering pneumatic resistance.

Samples were taken manually via drawing a volume of 10–15 mL during the end-tidal phase of an exhalation, as determined by CO_2_ measurements. Ultimately, a total of 500 mL of breath was collected from a single subject over 20–30 subsequent exhalations. Immediately after sampling, both ends of the sorbent tube were sealed with brass ¼” nuts, and the tubes were frozen at −80 °C. Samples were stored at −80 °C and transported on dry ice, with efforts made to minimize storage time to 4 weeks.

Relative standard deviations (RSDs) were calculated using 5 consecutively analyzed breath samples obtained from healthy volunteers. RSDs varied from 4 to 22%, which are considered adequate for the purposes of this study.

### 4.4. Gas Chromatography–Mass Spectrometry Examination of Breath Samples

A two-stage thermal desorption was performed using a thermal desorber and autosampler (TD100, Markes International Limited, Cardiff, UK). First, Tenax tubes were heated to 280 °C for 6 min under the constant flow of helium 6.0 (99.9999%) at 20 mL/min to desorb volatiles, that were next refocused in a cold trap packed with graphitized carbon black and maintained at 5 °C. The final injection of VOCs into the capillary column was achieved via the rapid heating of the cold trap to 320 °C for 1.5 min in a spitless mode.

The VOC separation and analysis were performed using an Agilent 7890A/5975C GC-MS system (Agilent, Santa Clara, CA, USA). Volatiles were separated using an Rxi-624Sil MS column (30 m × 0.32 mm, layer thickness 1.8 μm, Restek, Centre County, PA, USA) operated in constant helium flow of 1.5 mL min^−1^. The GC oven temperature program was as follows: 40 °C for 10 min, followed by 5 °C min^−1^ up to 150 °C, hold for 5 min, then 10 °C min^−1^ up to 280 °C, and isotherm at 280 °C for 5 min. The untargeted VOC analysis was performed using the mass spectrometer working in a SCAN mode with the associated *m*/*z* ranging from 20 up to 250. The peak integration was based on extracted *m*/*z* ratio chromatograms and such an approach allowed for the separation of the majority of peaks of interest from their neighbors. The quadrupole, ion source, and transfer line were kept at 150 °C, 230 °C, and 280 °C, respectively.

### 4.5. Statistical Data Analysis

Due to the deviation of VOC level values from a normal distribution, a non-parametric Wilcoxon rank-sum test was employed to assess and compare the measured VOC levels.

For this purpose, the breath gradient of the VOCs was used, i.e., the difference between the VOC level in breath and room air. A comparison was drawn between individuals with CRC and those without cancer, with significance set at a threshold of *p* < 0.05. Moreover, only VOCs with occurrences higher than 20% were taken into consideration. In this study, an untargeted analysis was performed to pinpoint volatile markers of CRC. Thus, the statistical analysis relied on the relative quantification and peak areas of detected metabolites were used as the parameter in the analysis. For the purposes of this study, limit of detection (LOD) was defined as three times the noise amplitude and only peaks with signal-to-noise ratio larger than 9 (3 × limit of quantification (LOD)) were taken into account.

## 5. Conclusions

VOCs are increasingly recognized as a valuable method for the early detection of a range of cancers, notably colorectal cancer (CRC). The aim of this study was to investigate and characterize the VOC chemical patterns associated with the breath of CRC patients and identify potential expiratory markers of this disease. The species emitted in higher amounts in CRC were p-xylene; hexanal; 2-methyl-1,3-dioxolane; 2,2,4-trimethyl-1,3-pentanediol disobutyrate; hexadecane; nonane; ethylbenzene; cyclohexanone; diethyl phthalate; 6-methyl-5-hepten-2-one; tetrahydro-2h-pyran-2-one; 2-butanone; benzaldehyde; dodecanal; benzothiazole; tetradecane; 1-dodecanol; 1-benzene; 3-methylcyclopentyl acetate; 1-nonene; toluene.

The results of this study provide compelling evidence that VOCs can be released in exhaled breath and serve as potential biomarkers for the presence of CRC. The identification of specific VOCs in the breath of CRC patients through GC-MS analysis offers valuable insights into the potential development of a breath-based diagnostic tool. Accurate identification of the VOCs linked to CRC is crucial for steering and refining the development of advanced sensor technologies. The distinct breath fingerprint associated with CRC holds promise for early detection and monitoring, presenting a non-invasive and patient-friendly approach to improving clinical outcomes. The findings underscore the significance of continued research in this field, as it is essential for translating these discoveries into robust and reliable diagnostic tools for CRC.

## Figures and Tables

**Table 1:** Gender and cancer stage and grade distribution in the study cohort.

		Cancer Group, n (%)	Control Group, n (%)	Total, n (%)	*p*-Value
Gender	Females	31 (44.0%)	40 (56.0%)	71 (100.0%)	0.836
	Males	47 (58.0%)	34 (42.0%)	81 (100.0%)	
Total included		78 (51.0%)	74 (49.0%)	152 (100.0%)	-
Cancer stage	I	16 (21.0%)	-	-	-
	II	37 (47.0%)	-	-	
	III	19 (24.0%)	-	-	
	IV	6 (8.0%)	-	-	
Cancer differentiation grade	1	23 (29.0%)	-	-	-
	2	43 (55.0%)	-	-	
	3	12 (15.0%)	-	-	

n—number of study participants.

**Table 2:** Volatile organic compounds categorized by chemical classes, with occurrence above 50%.

Chemical Class	Compound Name (CAS, Occurrence of Cancer/Non-Cancer (%))
Aldehydes	hexanal (66-25-1; 87/93), dodecanal (112-54-9; 73/82)
Esters	diethyl phthalate (84-66-2; 87/93)
Ketones	6-methyl-5-hepten-2-one, (110-93-0; 83/92), cyclohexanone (108-94-1; 74/76)
Hydrocarbons	hexadecane (544-76-3; 86/92), tetradecane (629-59-4; 81/84), nonane (111-84-2; 78/82), 1-nonene (124-11-8; 63/59)
Alcohols	1-butanol (71-36-3; 87/92), benzyl alcohol (100-51-6; 81/82), 1-dodecanol (112-53-8; 50/58)
Aromatics	ethylbenzene (100-41-4; 79/90), toluene (108-88-3; 85/88), p-xylene (106-42-3; 73/80), benzene (71-43-2; 73/80)
Heterocyclic	2-methyl-1,3-dioxolane (497-26-7; 58/54)

CAS—Chemical Abstracts Service.

**Table 3:** The list of breath compounds exhibiting differences between CRC patients and controls.

Compound Name	CAS	*p*-Value	Median (Q25,Q75) Breath Gradient		
			Level in CRC Group Compared to Control Group	Controls	CRC Patients
p-xylene	106-42-3	0.0005	↑	1,504,742 (936,665–2,127,623)	2,289,566 (1,418,724–3,064,362)
hexanal	66-25-1	0.0012	↑	1,091,070 (823,273–1,314,062)	1,369,804 (1,076,601–1,605,449)
2-methyl-1,3-dioxolane	497-26-7	0.0024	↑	331,109 (198,932–454,532)	484,592 (335,398–768,482)
2,2,4-trimethyl-1,3-pentanediol diisobutyrate	6846-50-0	0.0025	↑	1,030,684 (742,621–1,417,747)	1,341,632 (1,038,423–1,807,558)
hexadecane	544-76-3	0.0026	↑	838,697 (631,009–1,890,079)	1,598,483 (905,689–1,989,248)
nonane	111-84-2	0.0028	↑	354,633 (244,656–529,638)	484,690 (330,122–799,694)
ethylbenzene	100-41-4	0.0028	↑	357,817 (161,941–570,524)	596,334 (278,377–840,950)
cyclohexanone	108-94-1	0.0045	↑	164,463 (95,647–245,373)	210,847 (154,565–373,447)
diethyl phthalate	84-66-2	0.0060	↑	2,372,295 (1,244,073–4,668,721)	3,810,126 (2,186,709–6,749,612)
6-methyl-5-hepten-2-one	110-93-0	0.0076	↑	655,879 (349,775–1,046,236)	852,666 (581,498–1,227,127)
tetrahydro-2h-pyran-2-one	542-28-9	0.0093	↑	183,202 (159,649–232,003)	274,253 (198,146–419,828)
2-butanone	78-93-3	0.0109	↑	485,529 (338,060–609,519)	690,855 (445,338–901,846)
benzaldehyde	100-52-7	0.0126	↑	4721459 (3,876,629–5,921,829)	5,998,416 (4,588,636–7,960,831)
dodecanal	112-54-9	0.0127	↑	561,576 (413,502–769,154)	735,169 (519,829–876,106)
benzothiazole	95-16-9	0.0148	↑	158,701 (133,720–195,510)	199,243 (155,950–273,625)
tetradecane	629-59-4	0.0178	↑	1,098,813 (760,748–1,927,047)	1,521,759 (1,143,601–2,151,569)
1-dodecanol	112-53-8	0.0202	↑	544,799 (426,605–684,038)	712,005 (489,257–908,544)
benzene	71-43-2	0.0280	↑	1,148,309 (769,348–2,120,718)	1,830,674 (1,112,987–2,508,011)
3-methylcyclopentyl acetate	24070-70-0	0.0322	↑	319,377 (270,008–397,761)	406,271 (298,582–480,658)
1-nonene	124-11-8	0.0342	↑	247,535 (177,798–337,746)	318,057 (226,743–477,655)
toluene	108-88-3	0.0457	↑	1,192,237 (793,122–2,220,208)	1,553,576 (1,114,674–2,548,652)

CRC—colorectal cancer, CAS—Chemical Abstracts Service, Q25, Q27—interquartile range.

## Data Availability

The data presented in this study are available upon request from the corresponding author.

## Supplementary Materials

The following supporting information can be downloaded at: https://www.mdpi.com/article/10.3390/ijms25031632/s1.
