# Supplementary material for: Breath Fingerprint of Colorectal Cancer Patients Based on the Gas Chromatography–Mass Spectrometry Analysis
Source: Int J Mol Sci. 2024 Jan 29;25(3):1632. doi: 10.3390/ijms25031632 (PMC10855950; doi:10.3390/ijms25031632)
Supplement: Supplementary file 1 [file ijms-25-01632-s001.zip › ijms-2773487-supplementary.pdf]

Table S1. The full list of breath compounds exhibiting differences between colorectal cancer (CRC) patients and controls.

Compound name	CAS	*p-value*	Median Breath gradient		
			**Change**	**Controls**	**CRC patients**
p-xylene	106-42-3	0,0005	↑	1504742	2289566
hexanal	66-25-1	0,0012	↑	1091070	1369804
2-methyl-1,3-dioxolane	497-26-7	0,0024	↑	331109	484592
2,2,4-trimethyl-1,3-pentanediol diisobutyrate	6846-50-0	0,0025	↑	1030684	1341632
hexadecane	544-76-3	0,0026	↑	838697	1598483
nonane	111-84-2	0,0028	↑	354633	484690
ethylbenzene	100-41-4	0,0028	↑	357817	596334
cyclohexanone	108-94-1	0,0045	↑	164463	210847
diethyl phthalate	84-66-2	0,0060	↑	2372295	3810126
6-methyl-5-hepten-2-one	110-93-0	0,0076	↑	655879	852666
tetrahydro-2h-pyran-2-one	542-28-9	0,0093	↑	183202	274253
2-butanone	78-93-3	0,0109	↑	485529	690855
benzaldehyde	100-52-7	0,0126	↑	4721459	5998416
dodecanal	112-54-9	0,0127	↑	561576	735169
benzothiazole	95-16-9	0,0148	↑	158701	199243
tetradecane	629-59-4	0,0178	↑	1098813	1521759
1-dodecanol	112-53-8	0,0202	↑	544799	712005
1-butanol	71-36-3	0,0204	↑	593792	717135,5
benzene	71-43-2	0,0280	↑	1148309	1830674
3-methylcyclopentyl acetate	24070-70-0	0,0322	↑	319377	406271
1-nonene	124-11-8	0,0342	↑	247535,5	318057
toluene	108-88-3	0,0457	↑	1192237	1553576
1,2-benzenedicarboxaldehyde	643-79-8	0,0505	↑	5962967	6721031
o-xylene	95-47-6	0,0540	↑	324748,5	482457
n,n-dimethyl-methylamine	75-50-3	0,0572	↑	288222,5	364084
octane	111-65-9	0,0632	↑	331359,5	413934
3-hydroxy-4-methoxybenzaldehyde, tbdms	1000461-64-9	0,0668	↑	1774734	2032718
2-pentanone	107-87-9	0,0683	↑	675490	910249
1,2-ethanediol	107-21-1	0,0702	↑	209433	276367
undecanal	112-44-7	0,0726	↑	537808	606151
2-ethoxy-ethanol	110-80-5	0,0845	↑	472953	569275
isopropyl myristate	110-27-0	0,0876	↑	468005,5	1101405
2-heptanone	110-43-0	0,0893	↑	244945,5	347630
isoprene	78-79-5	0,0908	↑	57107655	70278739
1-pentanol	71-41-0	0,0922	↑	58115	89359

heptanal	111-71-7	0,1078	↑	1028365	1602589
acetic acid	64-19-7	0,1098	↑	6367227	8509098
2,4-dimethyl-1-heptene	19549-87-2	0,1107	↓	659368	544864
methyl-cyclohexane	108-87-2	0,1119	↓	728166	451208
dimethyl-silanediol	1066-42-8	0,1210	↓	911363	775272
acetoin	513-86-0	0,1219	↑	543455,5	766922
1-octene	111-66-0	0,1256	↑	264178,5	307074
acetone	67-64-1	0,1268	↑	79872400	88564930
nonanal	124-19-6	0,1350	↑	3512474	3641652
2-nonanone	821-55-6	0,1409	↑	197462	294991
phenylethyne	536-74-3	0,1446	↑	67835	87521
azulene	275-51-4	0,1480	↑	411085	457177
p-cresol	106-44-5	0,1487	↑	768100	1023809
heptane	142-82-5	0,1489	↑	711005	864525,5
decane	124-18-5	0,1635	↑	668251	714153
1-butoxy-2-propanol	5131-66-8	0,1671	↑	341984	434783
2-hexanone	591-78-6	0,1828	↑	151670,5	176214
pentanal	110-62-3	0,1880	↑	476091	513131
1,3,5,7-cyclooctatetraene	629-20-9	0,1906	↑	240164	324019
ethylidene-cyclopropane	18631-83-9	0,1943	↑	47931497	58528704
2-butoxy-ethanol	111-76-2	0,1952	↑	240105	281481
ethanol	64-17-5	0,2072	↑	1802018	2949049
tetramethyl-2,3,5,8-decane	192823-15-7	0,2109	↑	1193103	1567084
2-methyl-butane	78-78-4	0,2271	↑	590062	641142
benzeneacetaldehyde	122-78-1	0,2321	↑	373304	450221
decanal	112-31-2	0,2416	↑	2889239	2924331
2,3-dimethyl-pentane	565-59-3	0,2556	↓	164087	159752
1-ethyl-4-methyl-benzene	622-96-8	0,2766	↓	148937	137734
indole	120-72-9	0,2794	↓	1209785	994601
1-propanol	71-23-8	0,2871	↑	2020280	2350362
2-ethyl-1-hexanol	104-76-7	0,2916	↑	1838321	2036166
nonyl phenyl ester carbonic acid	1000314-57-3	0,3011	↑	739448	850432
5-ethyldihydro-2(3h)-furanone	695-06-7	0,3245	↑	68707	77532
isopropyl alcohol	67-63-0	0,3370	↓	67790453	45401775
acenaphthene	83-32-9	0,3699	↓	62682	57640
1,1'-oxybis-octane	629-82-3	0,3788	↑	376442	459867
methyl isobutyl ketone	108-10-1	0,3850	↓	199264	177063
p-cymene	99-87-6	0,3945	↓	445369	302998
d-limonene	5989-27-5	0,4170	↑	4097534	4312191

2-hydroxy-iso-butyrophenone	7473-98-5	0,4274	↑	674415	710831
1-nonanol	143-08-8	0,4371	↑	325692	350332
pentadecane	629-62-9	0,4445	↑	2038993	2208191
1-(methylthio)-propane	3877-15-4	0,4536	↑	446492	516631
3-heptanone	106-35-4	0,4803	↑	296116	308059
methyl vinyl ketone	78-94-4	0,5187	↑	1040030	1233134
dl-menthol	89-78-1	0,5290	↑	150085	176186
2-methyl-2-propanol	75-65-0	0,5325	↑	2208838	2792861
1-decanol	112-30-1	0,5353	↑	308903	348552
phenol	108-95-2	0,5712	↓	7981579	7323044
benzonitrile	100-47-0	0,5776	↑	558344	623925
biphenyl	92-52-4	0,6004	↑	79018	109412
1-octanol	111-87-5	0,6262	↑	532905	557608
2,3-butanedione	431-03-8	0,6603	↑	1090337	1231381
2-methyl-1,3,6-trioxocane	2781-01-3	0,6827	↑	431124	545008
heptadecane	629-78-7	0,6851	↓	801892	717741
allyl methyl sulfide	10152-76-8	0,7439	↑	589161	604135
tetrachloroethylene	127-18-4	0,7539	↓	192903	162847
tridecanal	10486-19-8	0,7581	↑	420528	422591
cyclohexane	110-82-7	0,7652	↑	579677	629151
n-hexane	110-54-3	0,7743	↑	154347	165048
butyl-cyclobutane	13152-44-8	0,7756	↑	121538	133191
1-methoxy-2-propyl acetate	108-65-6	0,7829	↑	286221	374213
dodecane	112-40-3	0,7829	↑	864686	868689
octyl ester octanoic acid	2306-88-9	0,8349	↑	500694	505542
4-methyl-octane	2216-34-4	0,8862	↑	184788	190877
2-methyl-1-propanol	78-83-1	0,9307	↓	1178750	1020187
3-methyl-hexane	589-34-4	0,9442	↑	324715	342228
tridecane	629-50-5	0,9626	↑	1582233	1653646
1-hexene	592-41-6	0,9821	↓	329981	311961
2,2,4,6,6-pentamethyl- heptane	13475-82-6	0,9854	↑	410893	443009
benzophenone	119-61-9	0,9967	↑	132838	135102
butanal	123-72-8	1,0000	↓	840323	804037
2,3-dimethyl-hexane	584-94-1	1,0000	↓	354395	329912
2-methyl-hexane	591-76-4	1,0000	↑	270492	284336
